# Supplementary material for: Brain-derived endothelial cells are neuroprotective in a chronic cerebral hypoperfusion mouse model
Source: Commun Biol. 2024 Mar 18;7:338. doi: 10.1038/s42003-024-06030-x (PMC10948829; doi:10.1038/s42003-024-06030-x)
Supplement: Supplementary file 2 — Supplementary Information [file 42003_2024_6030_MOESM2_ESM.pdf]

Supplementary Information

Article Title

Brain-derived endothelial cells are neuroprotective in a chronic cerebral hypoperfusion mouse model

Authors

Yuichi Matsui[1,2,#], Fumitaka Muramatsu[1,#], Hajime Nakamura[2], Yoshimi Noda[1],

Kinnosuke Matsumoto[1], Haruhiko Kishima[2] & Nobuyuki Takakura[1, 3, 4, 5*]

Division Organization

[1]Department of Signal Transduction, Research Institute for Microbial Diseases, Osaka University, Suita, Osaka, Japan

[2]Department of Neurosurgery, Osaka University Graduate School of Medicine, Suita, Osaka, Japan

[3] World Premier Institute Immunology Frontier Research Center, Osaka University, Osaka, Japan.

[4] Integrated Frontier Research for Medical Science Division, Institute for Open and Transdisciplinary Research Initiatives (OTRI), Osaka University, Osaka, Japan.

[5] Center for Infectious Disease Education and Research, Osaka University, Osaka, Japan.

[#] These authers contributed equally: Yuichi Matsui, Fumitaka Muramatsu.

Supplementary Methods

Assessment of blood–brain barrier function

To evaluate the retention of macromolecules in brain blood vessels, 155-kDa tetramethyl rhodamine (TRITC)-labeled dextran (125 mg/kg) was injected intravenously into the mice of a chronic cerebral hypoperfusion model on day 21. One hour after injection, the mice were euthanized and resected brains were fixed with 4% paraformaldehyde (PFA) in phosphate buffered saline (PBS).

Matrigel plug assay

C57BL/6 mice were anesthetized and endothelial cells (ECs; 2.5×10^5 cells) in 100 μL RPMI and Matrigel (Corning, Corning, NY, USA), mixed at 1:1, were injected subcutaneously into a flank. Matrigel plugs were collected after 7 days and fixed with 4% PFA in PBS for 1 hour at room temperature. Then, Matrigel plugs were quickly frozen in hexane cooled with liquid nitrogen and embedded in OCT compound.

Lenti-viral vector preparation

Mouse *Unc5b* cDNA was amplified by PCR and cloned into a pLVSIN-IRES-ZsGreen1 vector (Takara bio, Shiga, Japan). After primary ECs were cultured in medium containing a lentiviral vector for 3 hour, the medium was replaced and cells then cultured on OP9 feeder cells. The transfection of cells was determined by the presence of enhanced green fluorescent protein expression under a

fluorescence microscope.

Y-maze task

A Y-maze consisting of three arms (Brain Science Idea Co., Ltd., Osaka, Japan) was used in each Y-maze task test. Each arm was 40 cm in length, 15 cm in height, and 3 cm in width, with equal angles (named A, B, C). The behavioral assessments were performed according to a previous study[1]. Briefly, mice were placed in the center of a Y-maze and allowed to explore the arms freely for 8 minutes. The total number of entrances, the number of alternations or the count of sequential entrances in the three different arms (e.g., ABC, CAB, BCA…) were visually recorded. The percentage of each alternation was calculated as follows:

alternation behavior (%) = (number of alternations/total number of arm entrances-2) × 100

Supplementary References

1. Ghobadi, M. et al. Ferulic Acid Ameliorates Cell Injuries, Cognitive and Motor Impairments in Cuprizone-Induced Demyelination Model of Multiple Sclerosis. *Cell J.* **24**, 681-688 (2022)

Supplementary Figures

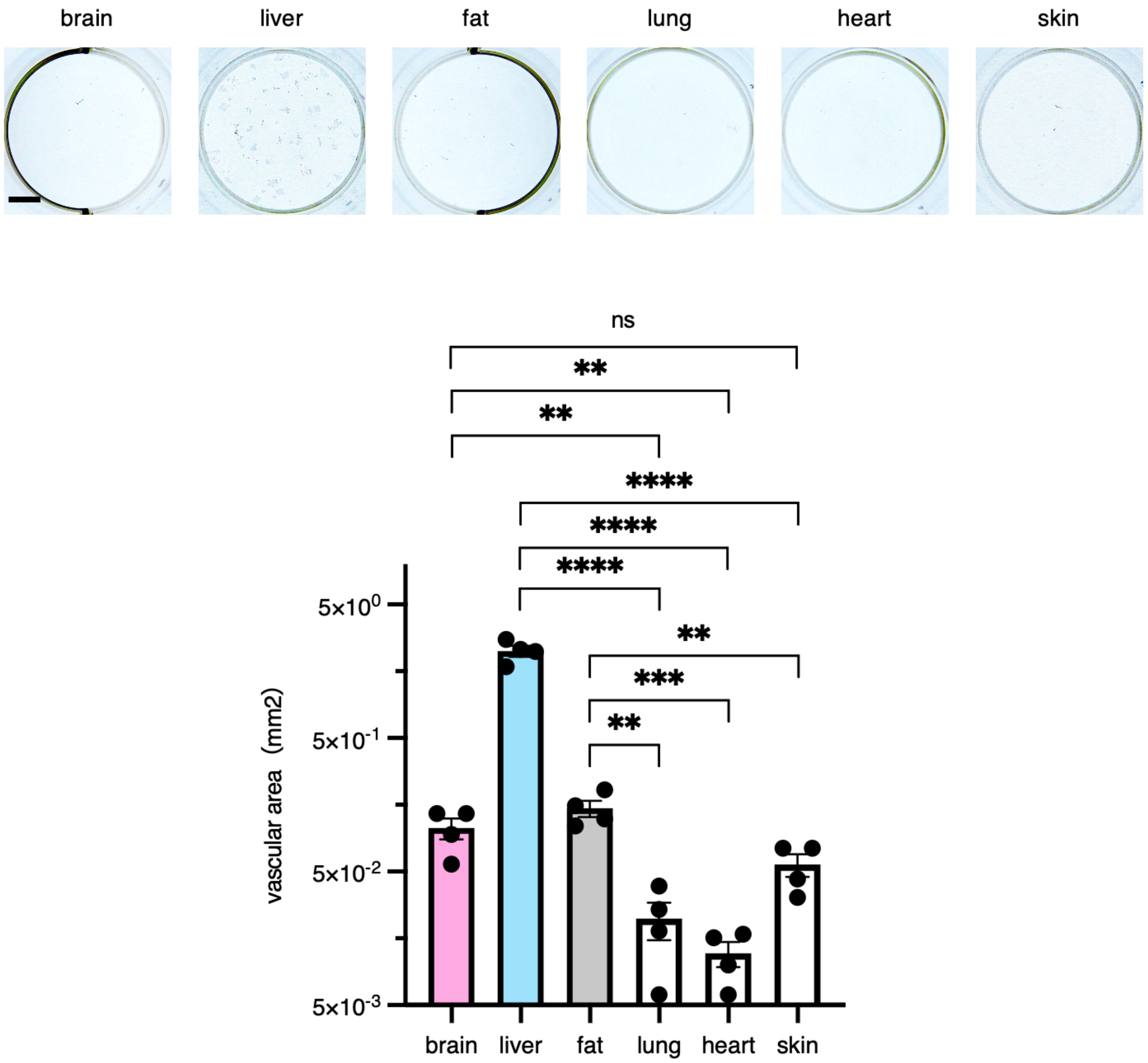

Supplementary Figure 1. Primary EC proliferation *in vitro*. ECs (5×10^3) isolated from each organ as indicated were cultured on OP9 feeder cells for 10 days and then stained with anti-CD31 antibody. Upper panels show representative photographs of EC colonies. Scale bar, 3 mm. The mean vascular area generated by ECs from each organ (n = 4 per group) was quantified. Data are present as mean ± SEM. **, $P < 0.01$; ***, $P < 0.001$; ****, $P < 0.0001$; EC, endothelial cell; ns, not significant

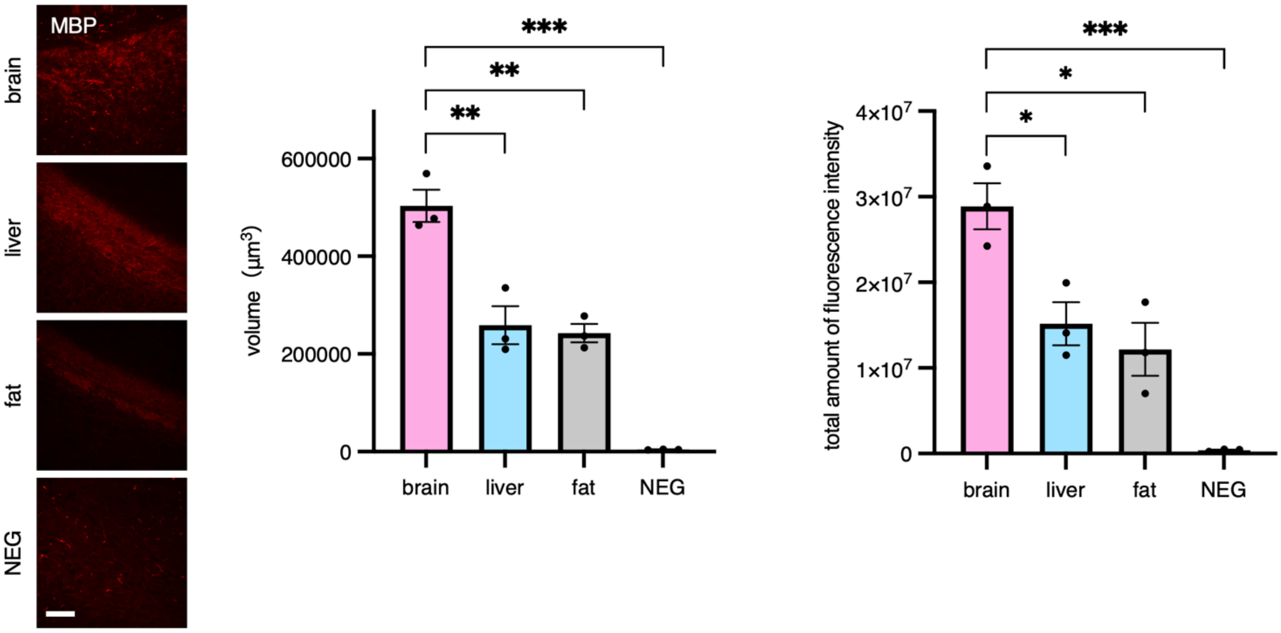

Supplementary Figure 2. Representative images of brain stained with anti-MBP polyclonal Ab (Alexa Flour 546) on day 28 after transplantation of ECs as described in Fig. 2. Scale bar, 50 μm. Bar graphs showing the mean volume and total amount of fluorescence intensity of MBP-positive cells (n = 3 per group), respectively, suggesting the degree of demyelination. Data are presented as mean ± SEM. *, $P < 0.05$; **, $P < 0.01$; ***, $P < 0.001$. EC, endothelial cell; MBP, myelin basic protein; NEG, negative control mice that did not receive cell transplants.

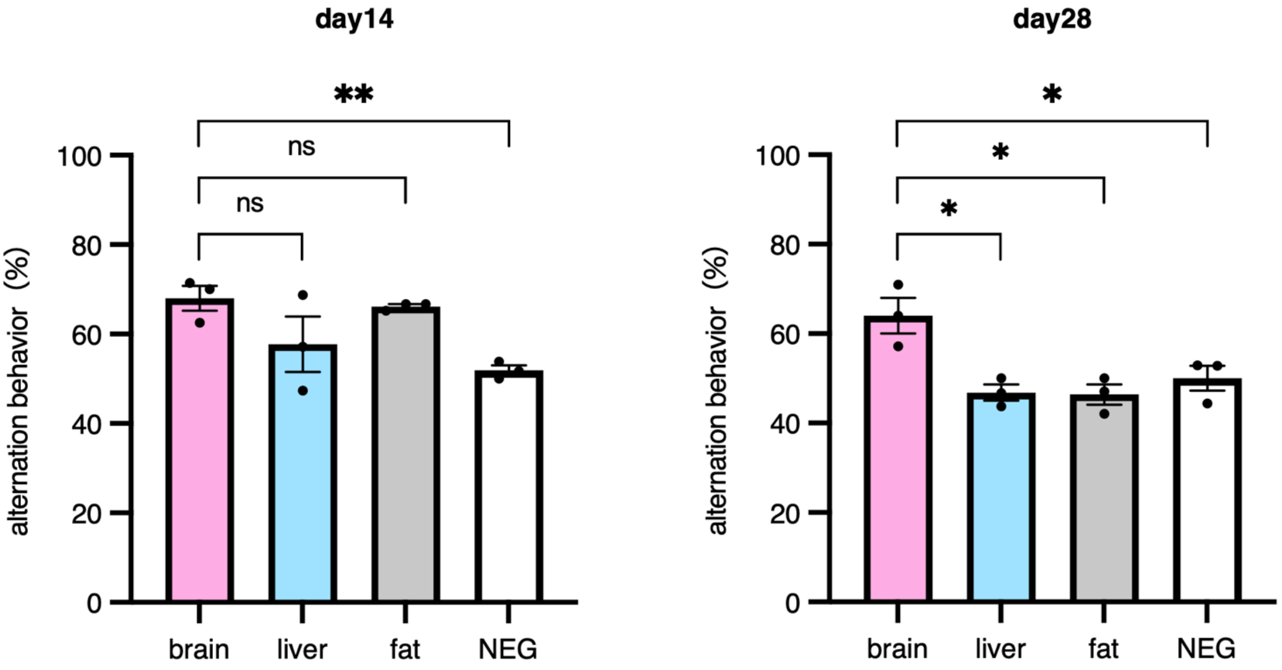

Supplementary Figure 3. Bar graph showing the mean alternation behavior of mice on days 14 and 28 (n = 3 per group). Mice were transplanted with ECs from various organs and Y-maze tests were performed. alternation behavior (%) = (number of alternations/total number of arm entrances-2) × 100. Data are presented as mean ± SEM. *, $P < 0.05$; **, $P < 0.01$; EC, endothelial cell; NEG, negative control mice that did not receive cell transplants; ns, not significant.

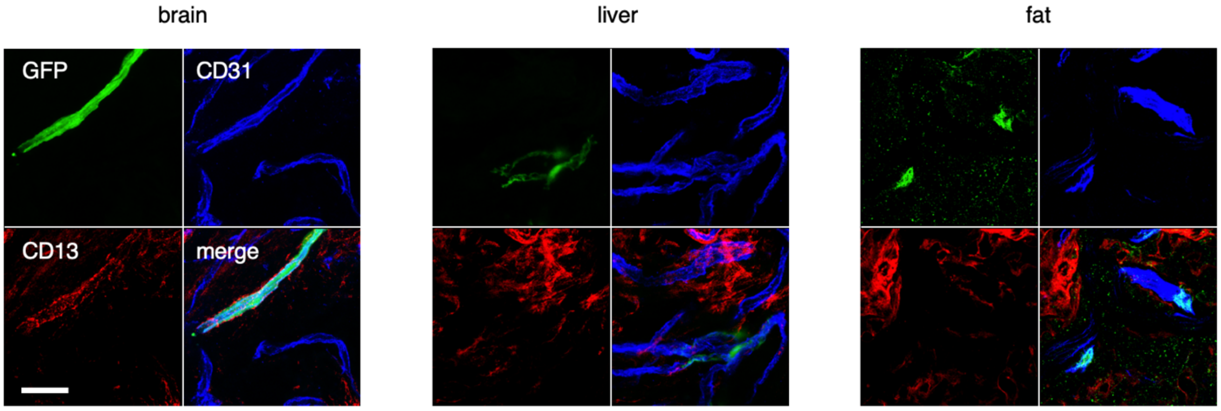

Supplementary Figure 4. Vascular phenotypes in brains transplanted with ECs from different organs were analyzed. Representative images stained with anti-CD31 mAb (Alexa Fluor 647) and anti-CD13 mAb (Alexa Flour 546) in regenerated vasculature areas transplanted with GFP-positive ECs derived from organs as indicated. Scale bar, 30 μm. EC, endothelial cell; GFP green fluorescent protein; mAb, monoclonal antibody

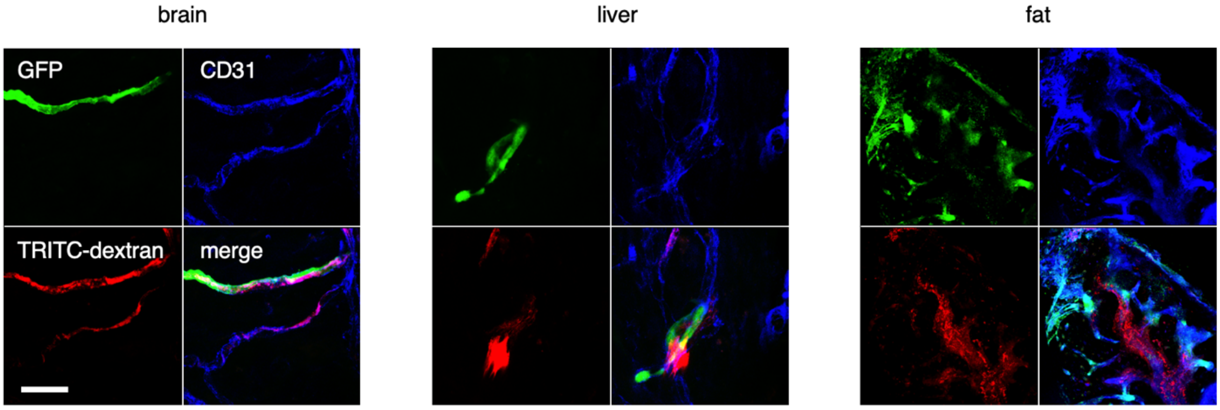

Supplementary Figure 5. Vascular phenotypes in brains transplanted with ECs from different organs were analyzed. To evaluate the retention of macromolecules in brain blood vessels TRITC-labeled dextran was injected intravenously into the mice of a chronic cerebral hypoperfusion model on day 21. One hour after injection, mice were euthanized and resected brains were fixed. Representative images showing staining with anti-CD31 mAb (Alexa Fluor 647) in regenerated vasculature transplanted with GFP-positive ECs derived from organs as indicated. Scale bar, 30 µm. EC, endothelial cell; GFP, green fluorescent protein; TRITC, tetramethyl rhodamine

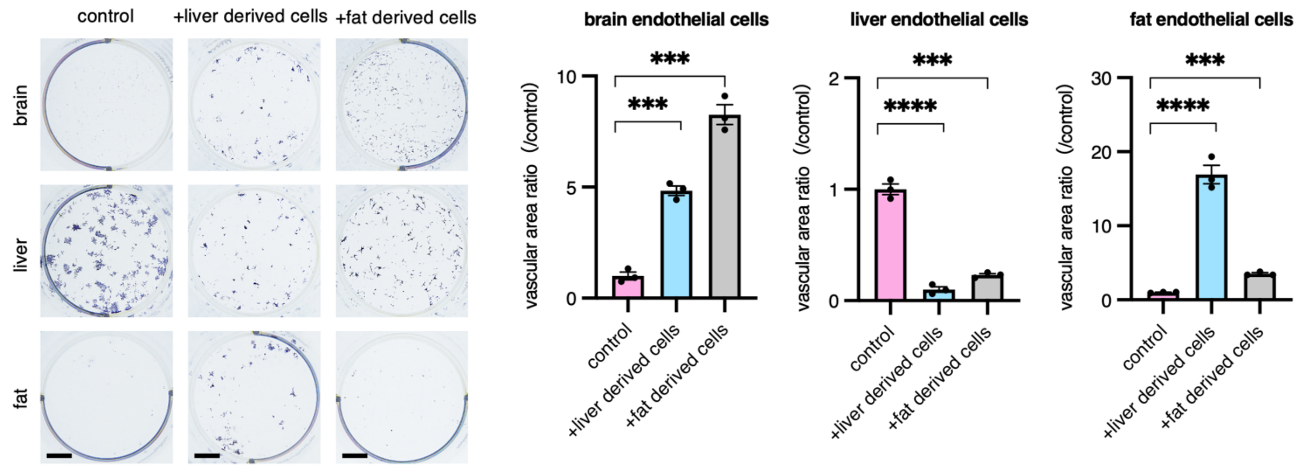

Supplementary Figure 6. EC proliferation *in vitro*. ECs (5 × 10^3) isolated from each organ as indicated were cultured with or without (control) liver- and fat-derived cells on OP9 feeder cells for 10 days and stained with anti-CD31 antibody. Left panels show representative photographs of EC colonies. Scale bar, 3 mm. The mean vascular area generated by ECs from each organ (n = 3 per group) was quantified relative to control. Data are presented as mean ± SEM. ***, $P < 0.001$; ****, $P < 0.0001$. EC, endothelial cell

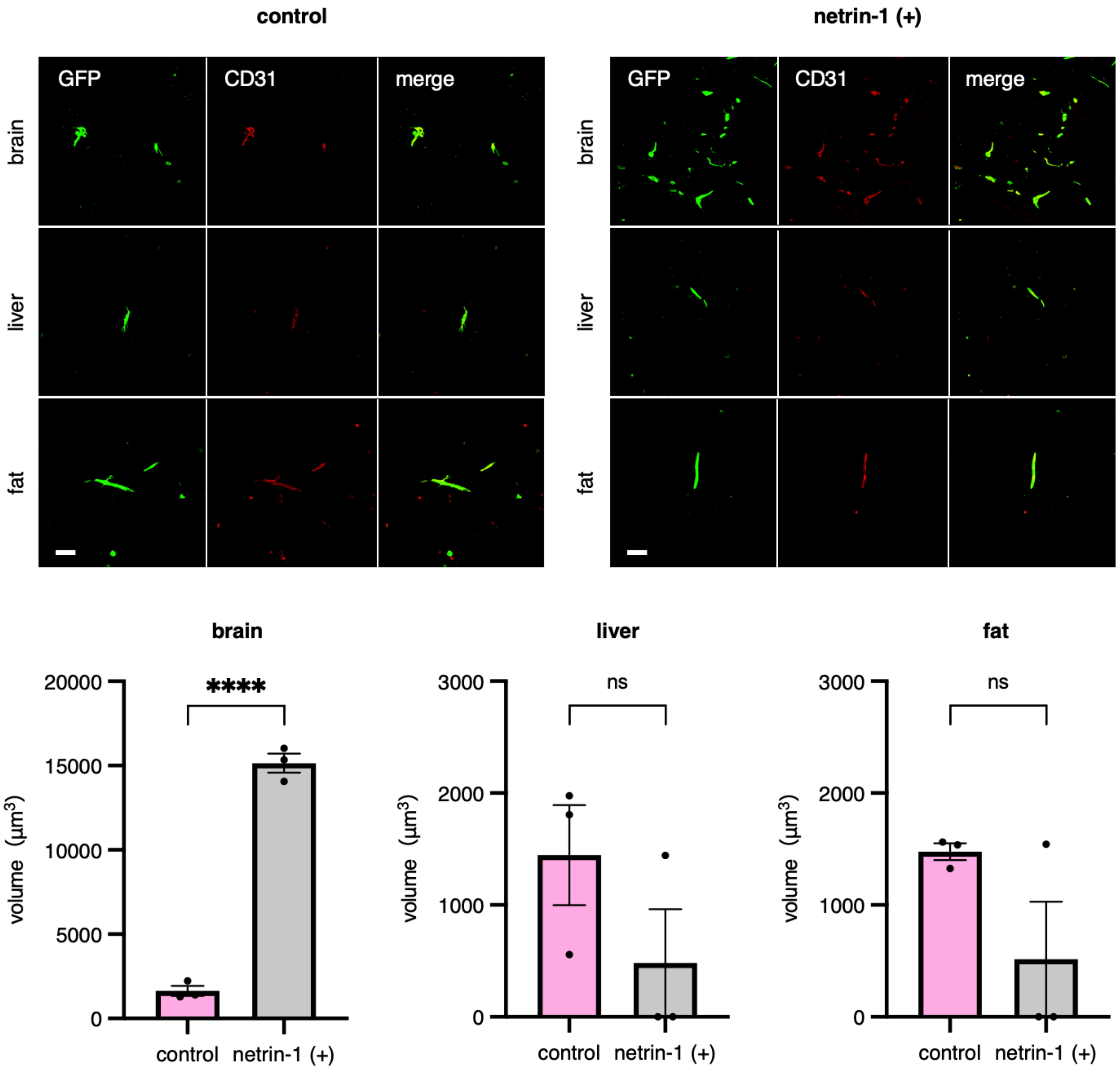

Supplementary Figure 7. ECs derived from various organs of GFP mice were mixed in Matrigel containing netrin-1 and injected subcutaneously into mice for a Matrigel plug assay. Upper panels show representative images stained with anti-CD31 mAb (Alexa Flour 546) in regenerated vasculature areas on day 7 after the injection of Matrigel. Scale bar, 50 µm. Bar graphs showing the mean volume of fluorescence intensity of GFP-positive cells (n = 3 per group). Data are presented as mean ± SEM. ****, $P < 0.0001$; EC, endothelial cell; GFP, green fluorescent protein; mAb, monoclonal antibody; ns, not significant.

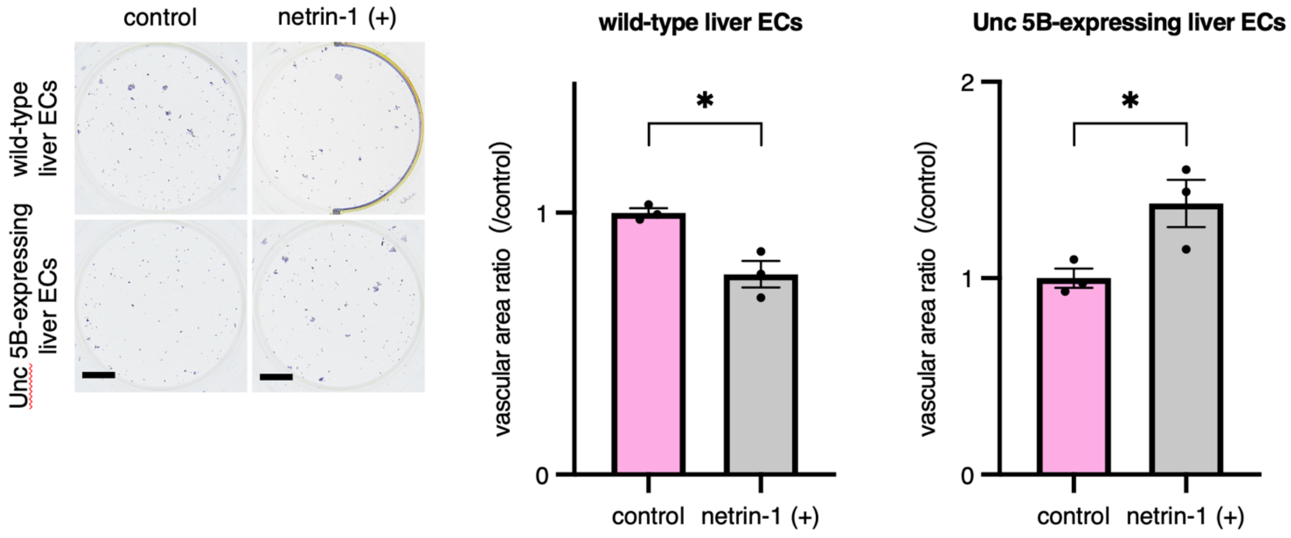

Supplementary Figure 8. EC proliferation *in vitro*. ECs (5×10^3) isolated from each organ as indicated were cultured on OP9 feeder cells for 10 days and stained with anti-CD31 antibody. Left panels show representative photographs of EC colonies. Scale bar, 3 mm. The mean vascular area generated by ECs from each organ (n = 3 per group) was quantified. Data are presented as mean ± SEM. *, $P < 0.05$. EC, endothelial cell

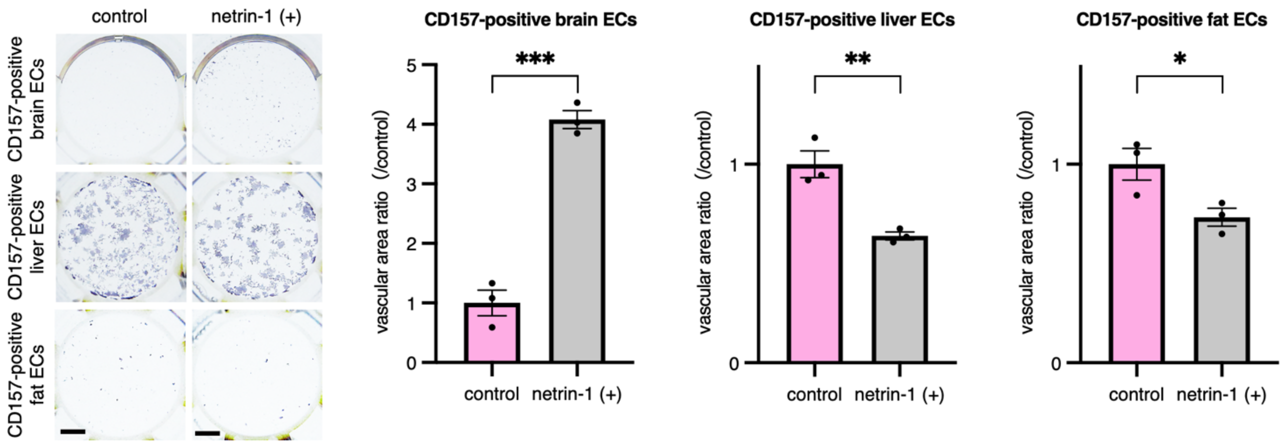

Supplementary Figure 9. EC proliferation *in vitro*. CD157$^+$ ECs (5×10^3) isolated from each organ as indicated were cultured on OP9 feeder cells for 10 days and stained with anti-CD31 antibody. Left panels show representative photographs of EC colonies. Scale bar, 3 mm. The mean vascular area generated by ECs from each organ (n = 3 per group) was quantified. Data are presented as mean ± SEM. *, $P < 0.05$; **, $P < 0.01$; ***, $P < 0.001$. EC, endothelial cell

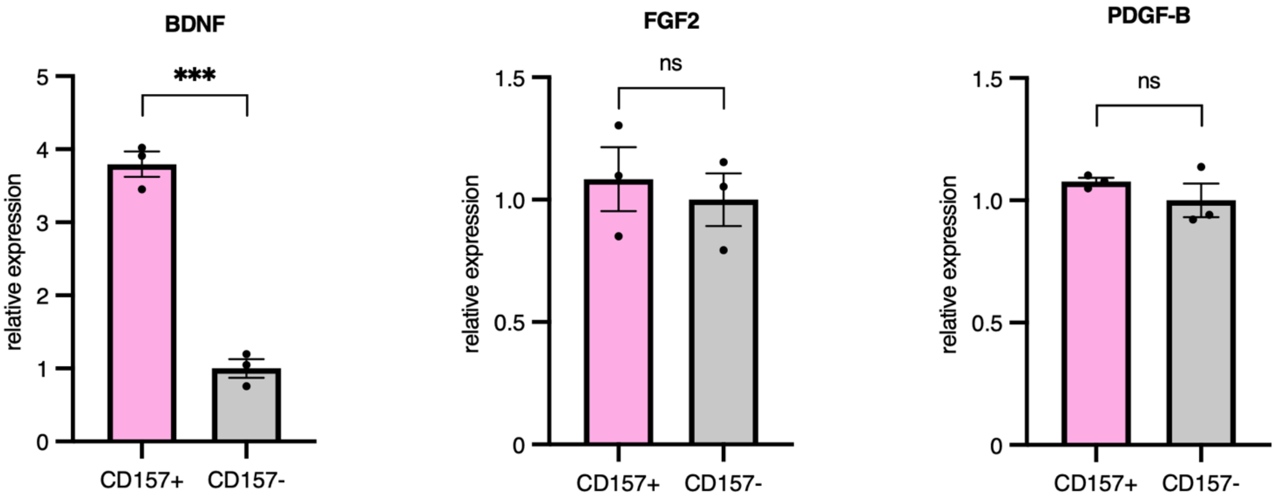

Supplementary Figure 10. Quantitative reverse transcription PCR analysis of neurotrophic and neuroprotective factors (BDFN, FGF2, and PDGF-B) in CD157-positive and CD157-negative ECs (n = 3 per group). Gene expression is relative to the gene expression level of CD157-negative ECs, which is set to 1. Data are present as mean ± SEM. ***, $P < 0.001$; ns, not significant. BDNF, brain-derived neurotrophic factor; EC, endothelial cell; FGF2, fibroblast growth factor 2; PDGF-B, platelet-derived growth factor-B.
